# Supplementary material for: Exploring replay
Source: Nat Commun. 2025 Feb 15;16:1657. doi: 10.1038/s41467-025-56731-y (PMC11829958; doi:10.1038/s41467-025-56731-y)
Supplement: Supplementary file 1 — Supplementary Information [file 41467_2025_56731_MOESM1_ESM.pdf]

Exploring replay

Supplementary Figures

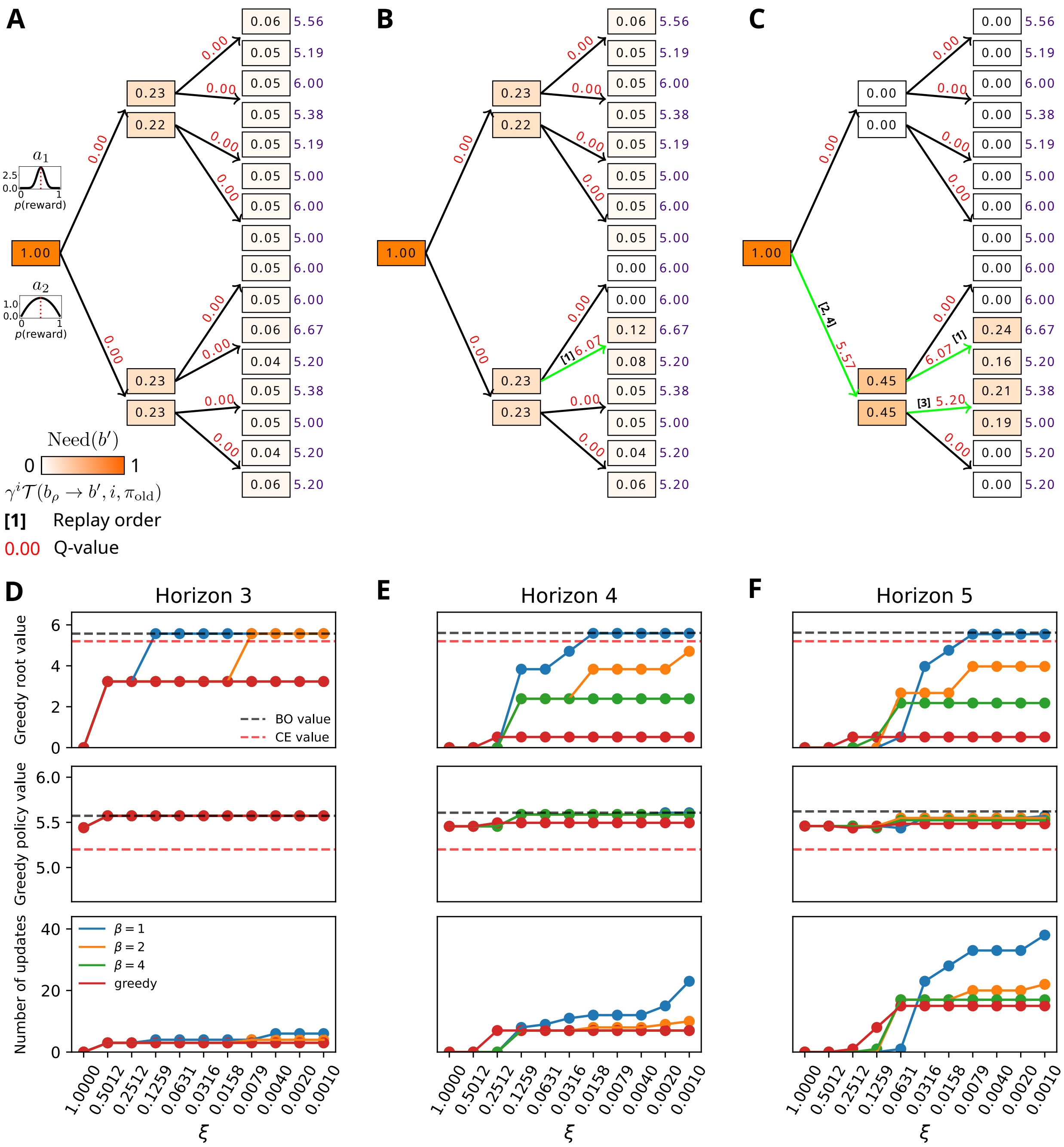

Supplementary Fig. 1: **Replay updates in Bandit belief space.** A) Planning tree of horizon 3. The layout of the figure is identical to that of Fig 2 except that the agent was uncertain about the payoff probabilities of both arms. Thus, each action resulted in two new belief states: one each for imaginary reward and non-reward. The prior belief state was initialised (shown with insets) such that the expected payoff probability for arm a_1 was equal to $\mathbb{E}_{p(\mu_1|b_\rho)}[\mu_1] = 0.52$, and the expected payoff probability for arm a_2 was equal to $\mathbb{E}_{p(\mu_1|b_\rho)}[\mu_1] = 0.50$, albeit with larger associated uncertainty. Notice that the replay updates shown in B)-C) chosen by the agent were concerned with arm a_2 which had a lower estimated immediate expected payoff probability. Such replay therefore produced a behavioural policy which was incentivised to explore the value of this uncertain arm. D)-F) show the effects of replay updates on the root and policy values of the agent as a function of the planning horizon and the replay threshold ξ, in the same manner as in Fig 2.

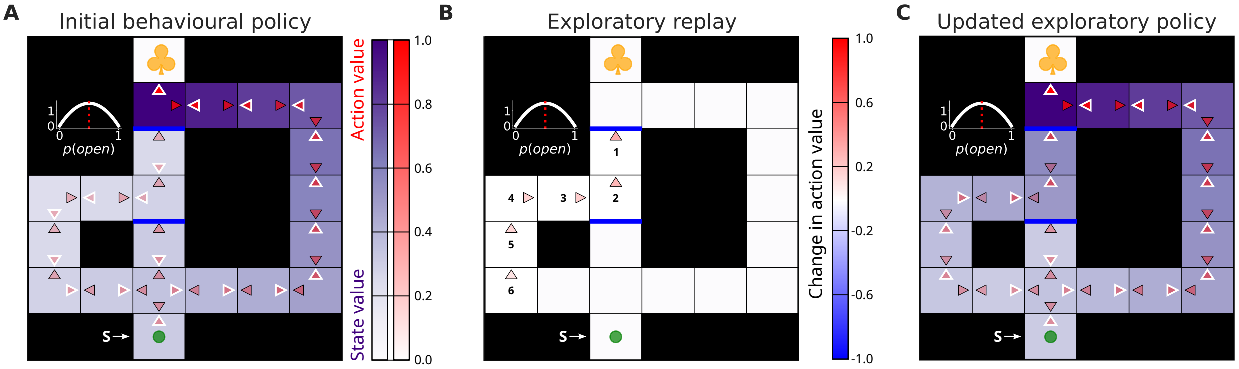

Supplementary Fig. 2: **Uncertainty affects replay choices and their behavioural readout.** The layout of the figure is similar to that of Fig 5. A) Prior state of knowledge of the subject. In this example, the subject's belief was more pessimistic since it indicated a lower subjective probability of the top barrier being potentially open (as evident from the expected probability the subject accorded to this possibility, shown with the red dotted line in the inset). B) The value of exploration was estimated to be lower (since the subject's belief was more pessimistic), and therefore replay did not propagate the benefit of exploration deep enough (towards the subject's location). This is in part owing to the temporal discounting which decays the benefit of exploration with travel distance. C) The updated policy still prescribed the subject to exploit the longer path (maximal Q-values at each state are again shown with white outlines), since the critical action at the junction between the different arms had not been updated by exploratory replay.

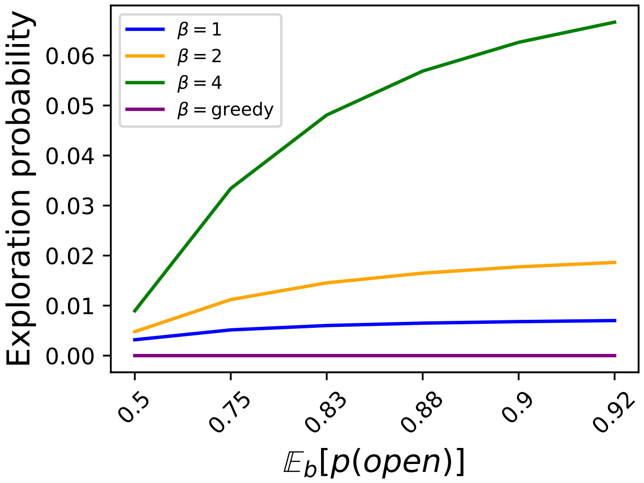

Supplementary Fig. 3: **Relationship between uncertainty, behavioural policy and exploration quality.** The graph shows the marginal probability of directed exploration (approaching and attempting the potential barrier in Figs 5 and 6 from the start state) as a function of the subject's uncertainty and the greediness of its behavioural policy. As the subject's belief ($\mathbb{E}[p(\text{open})]$) in the absence of the barrier increased, it became progressively more likely to engage in the act of directed exploration. Notice also that the greedy policy resulted in purely exploitative behaviour, since the agent's initial state of knowledge (that of Fig 5A) indicated that it should take the longest path, and therefore the exploratory Need for the potential exploratory replay updates was estimated to be 0.

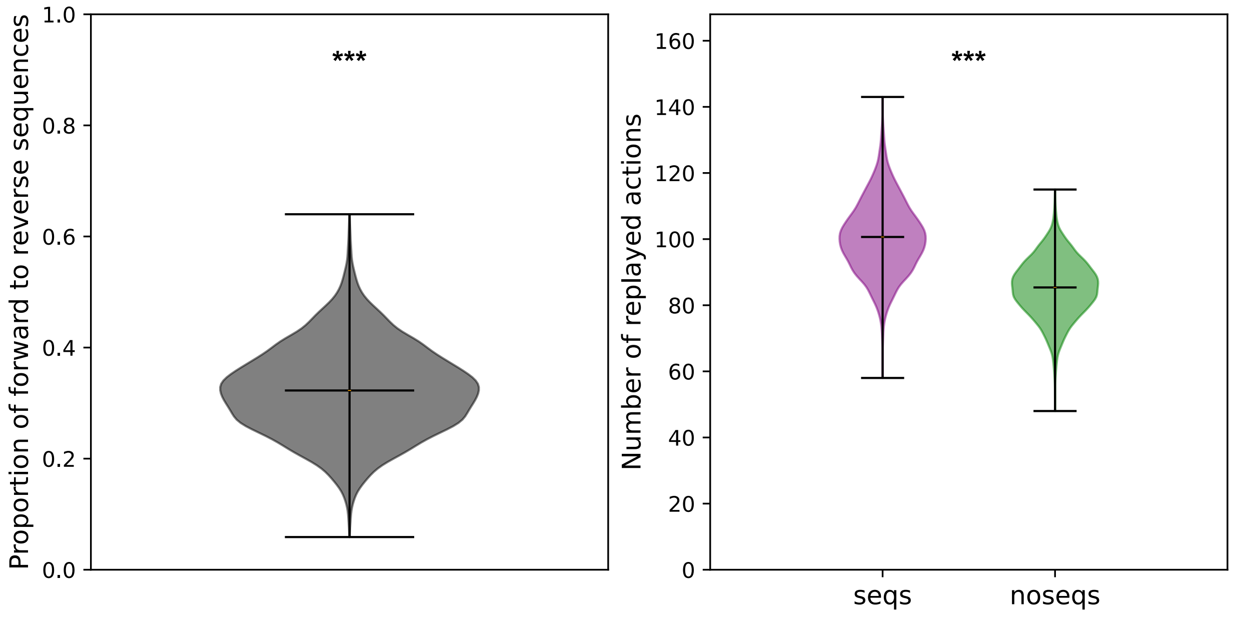

Supplementary Fig. 4: **Sequence replay statistics**. Left: violin plot showing the distribution of the proportion of forward to reverse sequences replayed in the belief tree in the MAB task with the same prior belief as in Supplementary Fig. 1 with planning horizon set to 4. The middle horizontal bar shows average proportion over 10000 different tree initialisations (the initialised values of all belief states in every tree were randomised). We found a significant proportion of forward sequences (1-sample 2-tailed t-test, $t = 403.54$, $p \ll 0.0001$), although with an evident bias towards reverse sequences. Right: same as above but for the average number of replayed actions in the same tree initialisations as with and without sequence replay. With sequences replay, significantly more actions received updates (2-sample 2-tailed t-test, $t = 110.33$, $p \ll 0.0001$). *** $p \ll 0.0001$. The orange bars show standard error of the mean and are too small to be visible.

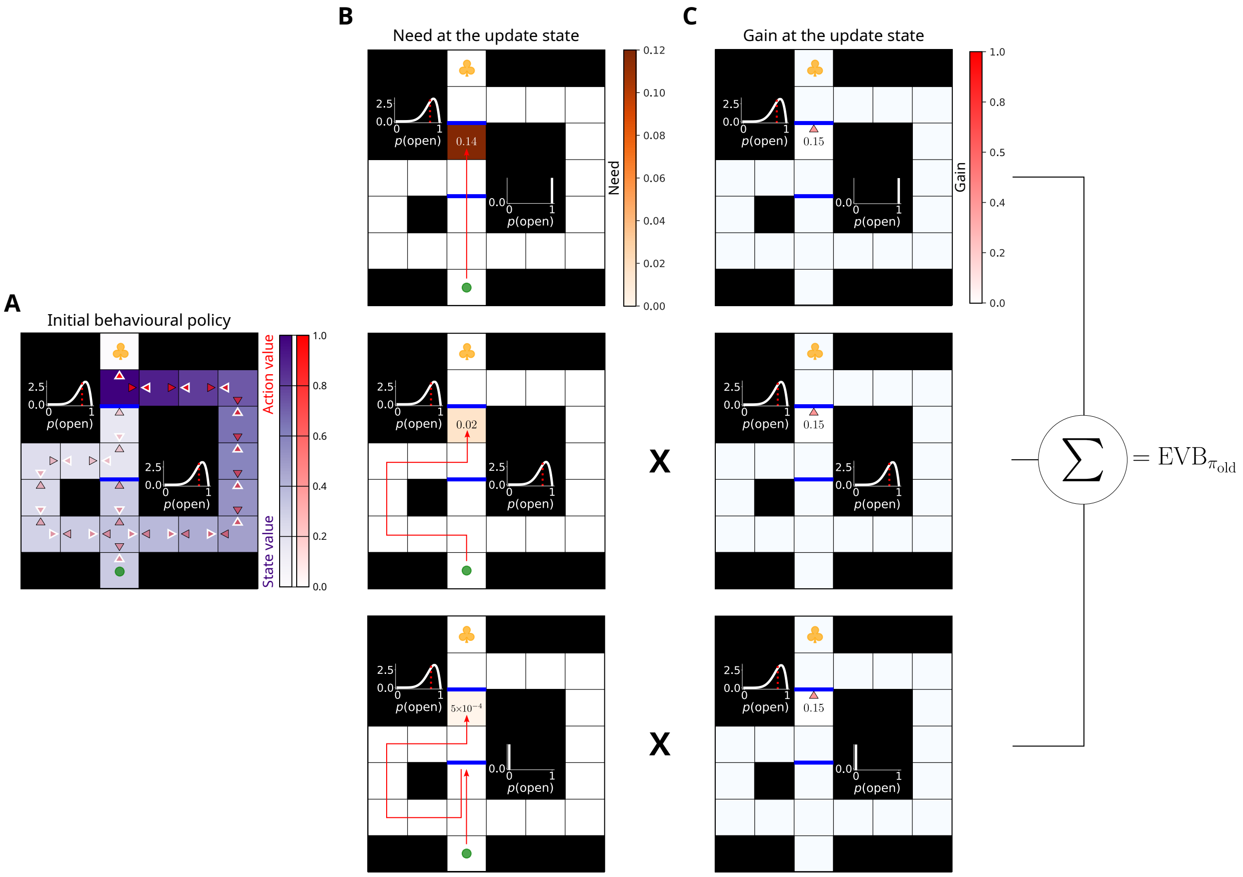

Supplementary Fig. 5: **The benefit of generalisation in replay across belief states**. A) Prior state of knowledge of the subject. The layout of the panel is identical to that of Fig 5. B) Need that the subject estimated for the potential update at the physical state below the top-most barrier. Each row shows a different belief with which the subject can reach that physical state. The red arrows denote the potential routes to that physical location that the agent can undertake all of which result in different belief states. For brevity, we only show a restricted number of the possible (discretised) beliefs. C) Estimated gain for the potential update of the action that attempts to cross the barrier. Note that Gain is positive in all the shown belief states associated with the top-most barrier. This means that the subject can expect to accrue more reward due to the update at that physical location whilst reaching it with different beliefs about the other (bottom) barrier. This knowledge of the potential future beliefs allows the subject to generalise across belief information states.

Supplementary Tables

Parameter	Value	Description
Q_{MF}^{init}	0	Initialised model-free Q-values
β	2	Inverse temperature
γ	0.9	Discount factor
h	2	Planning (replay) horizon
α_1, β_1	$1, 1$	Beta prior parameters for arm 1
μ_2	0.51	Known payoff probability for arm 2

Supplementary Table. 1: **Simulation parameters for Fig 1**.

Parameter	Value	Description
Q_{MB}^{init}	0	Initialised model-free Q-values
β	1	Inverse temperature
γ	0.9	Discount factor
h	3	Planning (replay) horizon
α_1, β_1	$1, 1$	Beta prior parameters for arm 1
μ_2	0.51	Known payoff probability for arm 2
ξ	0.0001	$\text{EVB}_{\pi_{\text{old}}}$ threshold

Supplementary Table. 2: **Simulation parameters for Fig 2**.

Parameter	Value	Description
Q_{MF}^{init}	0	Initialised model-free Q-values
α	1	Online learning rate
β	10	Inverse temperature
γ	0.9	Discount factor
ϕ_{MF}	0.05	Model-free forgetting
ξ	0.001	$\text{EVB}_{\pi_{\text{old}}}$ threshold

Supplementary Table. 3: **Simulation parameters for Fig 3**.

Parameter	Value	Description
β	2	Inverse temperature
γ	0.9	Discount factor
α_T, β_T	$7, 2$	Beta prior parameters for the top barrier
h	10	Planning (replay) horizon
N	2000	Number of simulated trajectories
ξ	0.000001	$\text{EVB}_{\pi_{\text{old}}}$ threshold

Supplementary Table. 4: **Simulation parameters for Fig 5**.

Parameter	Value	Description
β	2	Inverse temperature
γ	0.9	Discount factor
α_T, β_T	$7, 2$	Beta prior parameters for the top barrier
h	10	Planning (replay) horizon
L	8	Maximal sequence length
N	2000	Number of simulated trajectories
ξ	0.000001	$\text{EVB}_{\pi_{\text{old}}}$ threshold

Supplementary Table. 5: **Simulation parameters for Fig 6.**

Parameter	Value	Description
β	2	Inverse temperature
γ	0.9	Discount factor
α_T, β_T	$7, 2$	Beta prior parameters for the top barrier
α_B, β_B	$7, 2$	Beta prior parameters for the bottom barrier
h	6	Planning (replay) horizon
L	4	Maximal sequence length
N	2000	Number of simulated trajectories
ξ	0.000001	$\text{EVB}_{\pi_{\text{old}}}$ threshold

Supplementary Table. 6: **Simulation parameters for Fig 7.**
